# A comparison of objective structured clinical examinations (OSCEs) for medical students, modified during the COVID-19 pandemic

**DOI:** 10.1007/s11845-025-03976-7

**Published:** 2025-07-18

**Authors:** Andrew O’Malley, Nóirín Fitzgerald, Edina Moylett, Geraldine Gaffney, Geraldine McCarthy, Andrew W. Murphy, Rosemary Geoghegan, Brian Hallahan

**Affiliations:** 1 Department of Psychiatry, School of Medicine, University Hospital Galway, Galway, Ireland; 2 School of Medicine, University of Galway, Galway, Ireland; 3 Department of General Practice, National University of Ireland, Galway, Ireland; 4 Department of Paediatrics, University Hospital Galway, Galway, Ireland; 5 Department of Obstetrics and Gynaecology, University Hospital Galway, Galway, Ireland; 6 Sligo-Leitrim Mental Health Services, Sligo University Hospital, Sligo, Ireland

**Keywords:** Advanced clinical skills, COVID-19, Objective structured clinical examinations

## Abstract

**Introduction and aims:**

Objective structured clinical examinations (OSCEs) are an integral part of medical education assessment. The advanced clinical skills (ACS) OSCE examines clinical skills in psychiatry, general practice, obstetrics and gynaecology and paediatrics for fourth-year medical students at the University of Galway. This study compares results between the 2019 OSCE, and two subsequent OSCEs (2020 and 2021) modified to varying degrees secondary to the COVID-19 pandemic. We also examined student’s satisfaction and perspectives regarding both modified OSCEs.

**Materials and methods:**

Anonymised results between the 2019 (128 min), 2020 (56 min) and 2021 (96 min) ACS OSCEs were compared, and student feedback pertaining to the 2020 and 2021 OSCEs was analysed.

**Results:**

A higher total mean mark OSCE result was achieved at the 2020 OSCE (62.95%) compared to the 2019 (59.35%) and 2021 (58.89%) OSCEs (*F* = 31.83, *p* < 0.001), with significantly more first-class honours marks attained in 2020 (11.5%) compared to 2019 and 2021 (both 1%) (*p* < 0.001). Measures of reliability were consistent across all years. A majority of students in both 2020 and 2021 reported the OSCE to be safe, well-coordinated, and fair, but similar numbers of students from both 2020 and 2021 expressed concern that face masks impeded their communication skills.

**Conclusion:**

This study demonstrates the feasibility of conducting a modified reliable OSCE during a pandemic. Conducting a 96-min OSCE demonstrated similar results to the pre-COVID-19 pandemic 128-min OSCE, in contrast to a 56-min OSCE where potentially inflated marks were attained.

## Introduction

Objective structured clinical examinations (OSCEs) play a pivotal role in medical education and assist, evaluating the “show how” component in Miller’s pyramid of assessment [1]. OSCEs involve an assessment of student performance in a simulated clinical scenario (“station”) with a standardised patient with the aim of assessing skills such as collection and integration of clinical information and communication skills [2]. There is consistent evidence that OSCEs are valid and reliable assessment tools [3, 4], with this methodology frequently used to evaluate medical students’ suitability to progress onto medical qualification at both undergraduate and post-graduate levels [5, 6]. OSCE reliability depends on a number of factors including the number of stations, standardised scoring rubric, examiner training and standardised patient performance [7]. Optimising these factors influences the utility of a proposed examination as they influence important factors such as acceptability and cost [8]. 

In response to the onset of the COVID-19 pandemic in March 2020, medical education structures and assessments including OSCEs required adaptation to maintain feasibility. A recent systematic review identified two main responses internationally to adjusting OSCE delivery in the context of the COVID-19 pandemic, a wholly online OSCE delivery or a modified live OSCE format compliant with public health restrictions [9]. Whilst online OSCE delivery is feasible, with validity of assessment demonstrated [10, 11], concerns remain regarding this method’s suitability for assessing skills such as physical examination and procedural skills [12]. Modified live OSCE examinations have retained validity with a minimal risk of COVID-19 transmission where robust safety protocols are instituted [13, 14] although such OSCEs require very careful planning and significant circuit modification [15]. We described previously the adaptation of the fourth-year assessment of clinical skills (ACS) 2020 OSCE examination in response to this challenge at the University of Galway [13].

Although the adapted live OSCE conducted in 2020 was feasible, safe, with validity demonstrated (i.e. no difference in the overall pass rate compared to the previous year) [13], the examination encompassed a significantly shorter duration of examination time compared to pre-COVID-19 pandemic OSCEs (56 v. 128 min), with higher mean marks noted. As a higher number of OSCE stations is known to increase examination reliability [16], the fourth-year ACS committee adjusted the format for the 2021 examination with eight stations (two from each discipline—Psychiatry, General Practice, Obstetrics and Gynaecology and Paediatrics) of 12 min each (see Table 1). Some changes instituted due to the COVID-19 pandemic for 2020 were continued in 2021 (i.e. wearing of face-masks and strict hand hygiene), with other changes discontinued due to reduced governmental mandated public restrictions (i.e. segregation of examiners, temperature checks, travel declarations) (see Appendix 1).

**Table 1:** ACS OSCE grades

	2019 Mean (SD) (*n* = 200)	2020 Mean (SD) (*n* = 200)	2021 Mean (SD) (*n* = 205)	Statistics *F, p*
General practice	61.65 (6.91)	**69.03 (10.02)**	61.97 (6.33)	55.57, < 0.001
Obstetrics and gynaecology	56.35 (6.60)	**59.13 (8.99)**	57.89 (6.84)	6.70, 0.001
Paediatrics	61.76 (8.15)	62.44 (9.95)	**58.61 (7.84)**	11.25, < 0.001
Psychiatry	57.69 (6.08)	**61.22 (7.40)**	57.09 (5.66)	24.33, < 0.001
Total ACS mark	59.35 (5.54)	**62.95 (6.21)**	58.89 (4.93)	31.83, < 0.001
Cronbach’s alpha	0.78	0.80	0.75	
	***n* (%)**	***n* (%)**	***n* (%)**	*χ*^2^, *p*
ACS Grade				42.32, < 0.001*
H1	2 (1.0)	**23 (11.5)**	2 (1.0)	
H2	97 (48.5)	113 (56.5)	94 (45.9)	
Pass	93 (46.5)	58 (29.5)	101 (49.3)	
Fail	8 (4.0)	5 (2.5)	8 (3.9)	

*SD* standard deviation

^*^Fisher’s Exact test utilised

Our initial study compared the pre-pandemic OSCE of 2019 with that undertaken in 2020 during public health restrictions secondary to the pandemic (Fitzgerald et al., 2022). Our aim in this study was to compare data across the three different OSCEs to (1) compare results between the examinations for the entire OSCE and the discipline sub-components (Psychiatry, General Practice, Obstetrics and Gynaecology, and Paediatrics) in the context of OSCE modification in response to the COVID-19 pandemic and (2) assess student satisfaction with the modified OSCEs employed.

## Methods

Participants involved in this study included all 4 MB medical students sitting the ACS OSCE for the first time, in either 2019, 2020 or 2021. All data relating to exam performance were anonymised and securely stored and handled in accordance with the Data Protection Act, 2018. Ethical approval was attained from the Galway University Hospitals Research Ethics Committee (C.A. 2351).

Data pertaining to metrics from the three OSCE examinations, which is only available to members of the ACS committee, were retrieved from Observe software (Qpercom Ltd, 2020). The same previously validated anonymised 7-item questionnaire to examine students’ subjective experience of the modified OSCE was utilised for the 2020 and 2021 OSCEs (see Appendix 1).

Statistical analysis was conducted utilising the Statistical Package for Social Sciences 26.0 for Windows (SPSS Inc., IBM, New York, USA). Descriptive analyses (frequencies, means and standard deviations) were attained for all quantitative data (i.e. total OSCE result and scores attained from each OSCE station and each medical discipline) with data checked to ascertain if normally distributed. Mean marks were compared between years utilising analysis of variance (ANOVA) with grades attained compared utilising Chi Square (*χ*2) or Fisher’s exact test as appropriate. Likert scale data from this anonymized feedback were compared utilising the Mann–Whitney *U* test as data was not parametrically distributed. Free-text data were examined and open-coded based on the framework of the questionnaire. Data attained was then grouped into themes by consensus of the researchers (AOM, BH).

## Results

### OSCE metrics

Similar numbers of students undertook all three examinations (2019 = 200, 2020 = 200, 2021 = 205). The mean marks for the three OSCEs (including four sub-components) are presented in Table 1. Of note, the total mean mark was higher at the 2020 OSCE (62.95%) compared to the 2019 (59.35%) and 2021 (58.89%) OSCEs (*F* = 31.83, *p* < 0.001), with more first-class honours marks also attained in 2020 (11.5%) compared to 2019 and 2021 (both 1%) (*p* < 0.001). The overall OSCE and three disciplines (Psychiatry, General Practice and Obstetrics and Gynaecology) demonstrated a significantly higher mean mark for the 2020 examination compared to the 2019 and 2021 examinations (Tables 1 and 2). The 2021 OSCE demonstrated a similar overall mark compared with the pre-COVID 2019 OSCE, with only one discipline (Paediatrics) noting a lower mark (3.15%, *p* < 0.001) (post-hoc analysis presented in Table 2).

**Table 2:** ACS OSCE post-hoc comparisons between years

	Mean difference (95% CI)	*p*
General practice		
2021 v. 2019	0.32 (− 1.23, 1.87)	0.69
2021 v. 2020	− 7.06 (− 8.61, − 5.51)	< 0.001
2019 v. 2020	− 7.38 (− 8.93, − 5.82)	< 0.001
Obstetrics and gynaecology		
2021 v. 2019	1.54 (0.06, 3.01)	0.04
2021 v. 2020	− 1.22 (− 0.25, 2.70)	0.10
2019 v. 2020	− 2.76 (− 4.24, − 1.28)	< 0.001
Paediatrics		
2021 v. 2019	− 3.15 (− 4.85, − 1.46)	< 0.001
2021 v. 2020	− 3.83 (− 5.53, − 2.14)	< 0.001
2019 v. 2020	− 0.69 (− 2.49, 1.03)	0.43
Psychiatry		
2021 v. 2019	− 0.61, (− 1.86, 0.65)	0.35
2021 v. 2020	− 4.13 (− 5.39, − 2.88)	< 0.001
2019 v. 2020	− 3.53 (− 4.79, − 2.27)	< 0.001
Total ACS mark		
2021 v. 2019	− 0.48 (− 1.57, 0.61)	0.39
2021 v. 2020	− 4.06 (− 5.15, − 2.97)	< 0.001
2019 v. 2020	− 3.59 (4.68, − 2.49)	< 0.001

**Fig. 1:**
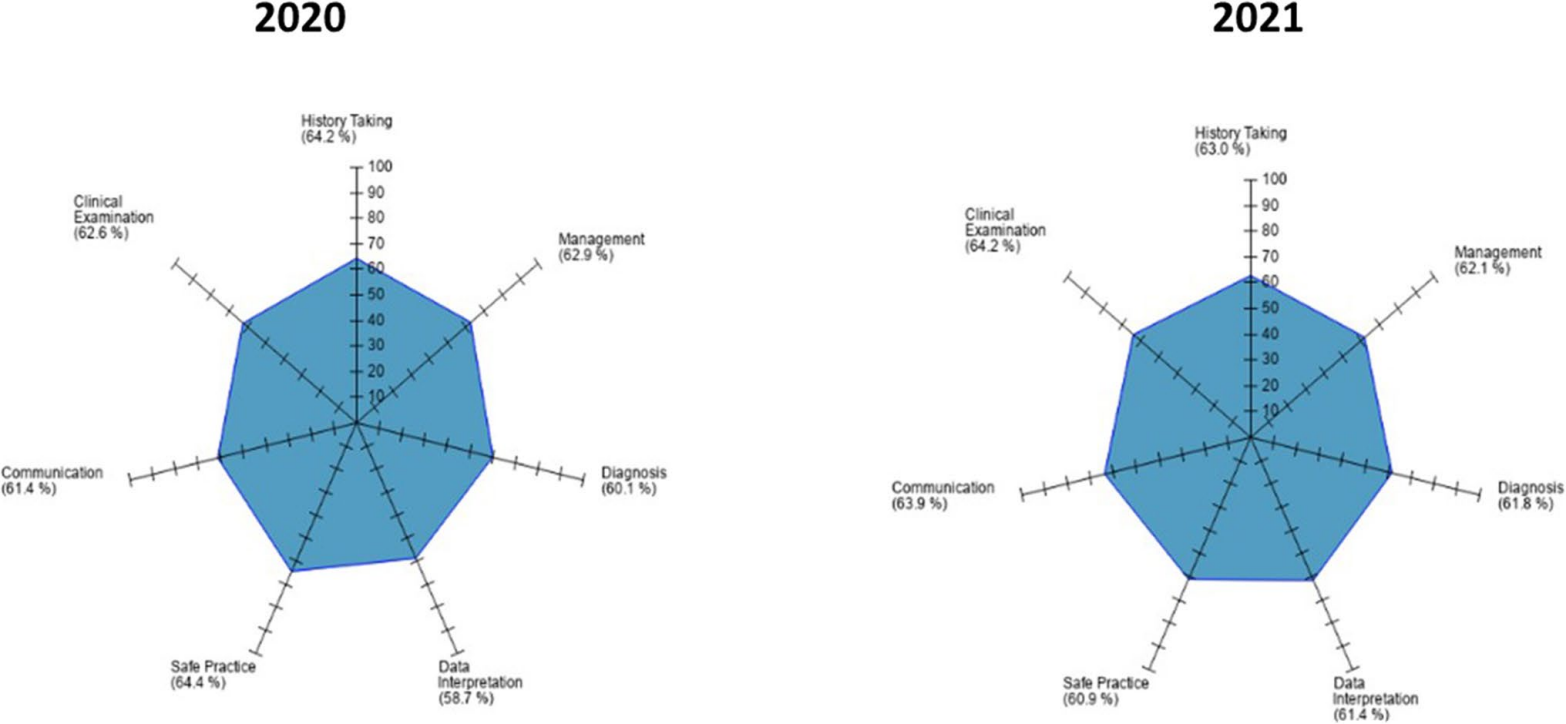
Spidergram of learning outcomes

The reliability of the OSCEs as measured utilising Cronbach’s alpha was similar across the three examinations. Spidergram data available for 2020 and 2021 noted similar scores for learning outcomes, with safety practice marks higher in 2020 compared to 2021 (64.4% v 60.9%) with communication skills higher in 2021 compared to 2020 (63.9% v 61.4%) (see Fig. 1).

### Student questionnaire

There was a lower response rate for the 2021 (*n* = 92, 30.7%) compared to 2020 (*n* = 92, 45.5%) anonymous student experience survey (Appendix 2). A majority of respondents at both OSCE sittings stated that they felt that the examination was safe (2020 = 89.1%, 2021 = 96.8%), well coordinated (2020 = 88.8%, 2021 = 88.9%)), with clear communication provided pre-OSCE (2020 = 61.5%, 2021 = 66.7%) and executed fairly (2020 = 61.3%, 2021 = 61.9%) (Table 3). A similar percentage of participants at both OSCEs felt that face masks impacted their ability to communicate with patients (2020 = 47.8%, 2021 = 52.4%). A higher percentage of participants were neutral in relation to their examination feedback in 2021 (78.0% v. 19.6%), perhaps reflecting that students either had not received or had not checked their feedback at the time of the questionnaire completion in 2021, with free-text data supporting same.

**Table 3:** OSCE: student feedback

	Strongly agree		Agree		Neutral		Disagree		Strongly disagree				Statistics	
	2020 *n* (%)	2021 *n* (%)	2020 *n* (%)	2021 n (%)	2020 *n* (%)	2021 *n* (%)	2020 *n* (%)	2021 *n* (%)	2020 *n*(%)	2021 *n*(%)			*U*, *p*	
Q1. I felt safe during the OSCE	**60 (65.2)**	**46 (73.0)**	22 (23.9)	15 (23.8)	6 (6.5)	2 (3.2)	2 (2.2)	1 (1.6)	2 (2.2)		1 (1.6)		3185.0, 0.84	
Q2. The OSCE was executed fairly	**29 (31.2)**	12 (19.0)	28 (30.1)	**27 (42.9)**	13 (15.1)	15 (23.8)	15 (16.1)	6 (9.5)	7 (7.5)		3 (4.8)		2778.0, 0.65	
Q3. The OSCE was well co-ordinated	**48 (52.2)**	**32 (50.8)**	34 (36.6)	24 (38.1)	6 (6.5)	3 (4.8)	3 (3.3)	2 (3.2)	1 (1.1)		2 (3.2)		2851.0, 0.85	
Q4. Communication received prior to the OSCE was clear and effective	28 (30.4)	11 (17.5)	**29 (31.5)**	**31 (49.2)**	16 (17.4)	14 (22.2)	16 (17.4)	6 (9.5)	3 (3.3)		1 (1.6)		2838.0, 0.82	
Q5. Spidergram feedback was useful and effective*	18 (19.6)	1 (1.7)	21 (22.8)	6 (10.2)	18 (19.6)	**46 (78.0)**	**25 (27.2)**	2 (3.4)	10 (10.9)		4 (6.8)		2553.0, 0.52	
Q6. Learning outcomes were clearly outlined and easily understood	13 (14.1)	6 (9.5)	**44 (47.8)**	**23 (36.5)**	10 (10.9)	16 (25.4)	20 (21.7)	13 (20.6)	5 (5.4)		5 (7.9)		2511.5, 0.14	
Q7. Wearing a face-mask affected my ability to communicate with patients during the OSCE	15 (16.3)	8 (12.7)	**29 (31.5)**	**25 (39.7)**	16 (17.4)	8 (12.7)	26 (28.3)	18 (28.6)	6 (6.5)		4 (6.3)		2912.5, 0.96	

U = Mann–Whitney *U* test. Respondents: 2020 = 92, 2021 = 63

^*^Five respondents who sat the OSCE in 2021 did not answer this question. It is likely that some participants completed the questionnaire prior to the attainment of this feedback

### Questionnaire: free-text data 2021

In total, four themes emerged relating to the 2021 OSCE: (1) experienced as too long in duration (*n* = 9), (2) a lack of examination feedback (*n* = 9), (3) a preference for examiners to provide more support to students during the OSCE (*n* = 6), and (4) that the OSCE was well organised (*n* = 3) (Box 1). Unlike in 2020 (Fitzgerald et al., 2022), there was no written feedback relating to the deleterious impact of mandated COVID-19 restrictions on OSCE performance, with feedback from the 2020 OSCE noting a preference for more OSCE stations, which was in contrast to the 2021 OSCE feedback.

**Table:** **Box 1** Themes collated from free-text student survey responses in 2021

Theme 1: The examination was too long in duration. N=9
• “During latter half of the OSCE I found myself losing concentration and making mistakes that I don’t think I would have made had there not been so many stations.” (#53)
• “I think having all 4 modules on one day was very overwhelming, as we had to know each one in detail as well as it being an exhausting two hours.” (#16)
Theme 2: Spidergram feedback not provided. N=9
• “We are yet to receive the spidergram feedback so I was not able to comment on that.” (#6)
• “Haven’t received feedback yet, but I think any feedback will be of great benefit.” (#58)
Theme 3: Examiners could give more support during the examination. N=6
• “From discussing with other classmates it seems the examiners were inconsistent e.g. some would correct you if you made a mistake whereas others would let you continue on the station incorrectly.” (#38)
• “Some of the examiners were not helpful during the stations. Like they see a student struggling but failed to prompt.” (#31)
Theme 4: The examination was well organised. N=3
• “It was very well run on the day.” (#32)
• “Overall the exam was well run and organized.” (#38)

## Discussion

This study demonstrated that mean grades in the 8 station 2021 ACS OSCE (96 min) were similar to those in the 16 station unmodified 2019 OSCE (128 min). In contrast, the modified 4 station 2020 OSCE (56 min) was associated with a 3–4% higher mean mark, with more first-class honours marks compared to the 2021 or 2019 OSCEs. Overall student feedback was positive regarding both the coordination of the modified OSCE, with similar feedback noted in 2020 and 2021; however, approximately half of the students from both OSCEs reported that wearing a face mask impeded their ability to communicate with the standardised patients during the OSCEs (albeit not reflected in marks attained).

There are a number of potential reasons which may attribute to the higher marks noted in the 2020 modified OSCE compared to both the 2019 and 2021 OSCEs. Firstly, students had a longer timeframe to prepare for the 2020 OSCE (as this examination was postponed from May until August), with additional teaching sessions conducted throughout the summer. Secondly, a reduction in the assessment time and stations being assessed by each discipline was associated with a reduced assessment blueprint (see Table 4). This resulted in all core features being assessed (i.e. risk and communication) but some clinical knowledge being assessed in less detail than with previous examinations (i.e. therapeutics), potentially making some components of the modified OSCE easier for some students [7, 17]. The 2021 OSCE examined all core features to a greater extent than the 2020 OSCE (37 v 18 LOs) but not to the extent of the 2019 OSCE (68 LOs), but this study suggests these core features were examined sufficiently in 2021, which perhaps was not the case in 2020 (see Table 4). Thirdly, the phenomenon of grade inflation as a consequence of the COVID-19 pandemic has been consistently documented in both second and third level education in Ireland and is another potential factor to be considered regarding this data [18].

**Table 4:** Examination blueprint with learning outcomes

Learning outcomes			2019				2020				2021			
			Paediatrics	Obgyn	GP	Psychiatry	Paediatrics	Obgyn	GP	Psychiatry	Paediatrics	Obgyn	**GP**	**Psychiatry**
On successful completion of the module the learner will be able to:		Domain												
1	Take a history from people of relevant specialties, across a wide range of different scenarios, showing a patient-centred, sensitive, multicultural, structured and thorough approach with demonstration of principles of good communication	**History taking**	**xxx**	**x**	**xxxx**	**xx**	**x**		**x**	**x**	**xx**		**xx**	**xx**
2	Undertake a physical examination/mental state examination that are systems-based; appropriate for patient’s age, gender and state of mental and physical health, in a rigorous, sensitive, efficient and systematic manner	**Clinical examination**	**x**	**xx**	**xx**	**xxx**		**x**	**x**		**x**	**xx**	**x**	**xx**
3	Demonstrate awareness of accepted professional attitude and behaviour with patients, carers and colleagues	**Communication**	**xxx**	**xxxx**	**xxxx**	**xxx**	**x**	**x**	**x**	**x**	**x**	**xx**		**xx**
4	Demonstrate awareness of patient safety in the specialist areas of Child Health, Women’s Health, Community and Mental Health	**Safe practice**	**xx**	**xxxx**	**xx**	**xxx**		**x**	**x**		**x**	**xx**		**x**
5	Evaluate and analyse common investigative test results, and interpret any positive or negative findings therein, and exhibit a further ability to request further appropriate investigations, in the specialty subjects	**Data interpretation**	**x**	**xx**	**x**	**x**	**x**		**x**		**x**	**xx**	**x**	
6	Synthesise competently, in the specialist clinical context, all available information gathered from history, examinations and basic investigate testing and formulate a reasonable working diagnosis and differential diagnosis, whilst recognising life threatening conditions that require immediate treatment	**Diagnosis**	**xx**	**xx**	**xxx**	**xx**		**x**	**x**		**x**	**xx**	**x**	**xx**
7	Explain effectively the diagnosis/prognosis and agree a management plan with the patient or team member, including reference to appropriate additional sources of expertise and information	**Management**	**xxx**	**xx**	**xxxx**	**xxx**		**x**	**x**	**x**	**x**	**x**	**xx**	**xx**

**x** = Learning outcome examined in OSCE station

Of note, markers of internal reliability of the examinations such as Cronbach’s alpha have remained relatively stable throughout the 3 years studied, indicating that examination reliability appears not to have been impacted, despite some relatively minor result variability. This is a particular strength of this study given that a recent systematic review noted that few studies on health professions assessment modification secondary to COVID-19 restrictions included measures of internal reliability, including none that were pertaining to OSCEs specifically [12]. However, our data suggests that the discriminative power of the OSCE regarding different grading categories (1st and 2nd class honours and pass grades) was significantly reduced in the 2020 OSCE, with a reduced OSCE blueprint being a potential factor.

This study is consistent with other international studies noting that modified OSCEs in response to COVID-19 pandemic restrictions are feasible and acceptable to students [10, 11], albeit restructuring circuits and amending OSCE venues are required to create an appropriate and safe environment [15, 19]. The time duration suggested for adequate reliability of an OSCE is quite broad (70–160 min) [16, 20, 21]. Of note, the modified 2021 OSCE duration of 96 min is identical to the median OSCE testing time across 11 medical schools in Australia in 2020 [22].

Both modified OSCEs required students to wear face masks, with questionnaire data noting concerns that these impeded communication with patients during the examination. However, free-text data in 2021 (but not in 2020) did not reflect these concerns, with spidergram feedback noting no deleterious impact of scores for communication skills (Fig. 1), suggesting that this domain can be assessed effectively in the presence of mandatory face masks despite student concerns. The modified OSCEs were associated with high levels of satisfaction with the organisation and coordination of the examination (i.e. survey and free-text responses), with students feeling safe. To our knowledge, no students or staff contracted COVID-19 as a result of attending the examination.

This study has a number of limitations. Firstly, no formal feedback was attained from the administrative staff and examiners involved in the preparation, organisation and marking of this examination. Secondly, we do not have feedback from students for the 2019 OSCE as this was only introduced in 2020, and response rates for feedback were relatively low, particularly in 2021.

## Conclusion

This study has demonstrated the feasibility of conducting a modified OSCE during a pandemic. This study suggests that an 8 station (96 min) OSCE demonstrated very similar results to the pre-COVID-19 pandemic 16 station (128 min) OSCE, in contrast to a 4 station (56 min) OSCE where overall marks and first-class honour grades were higher. High levels of reliability were noted for all three OSCEs.

## Figures and Tables

**Fig. 2:**
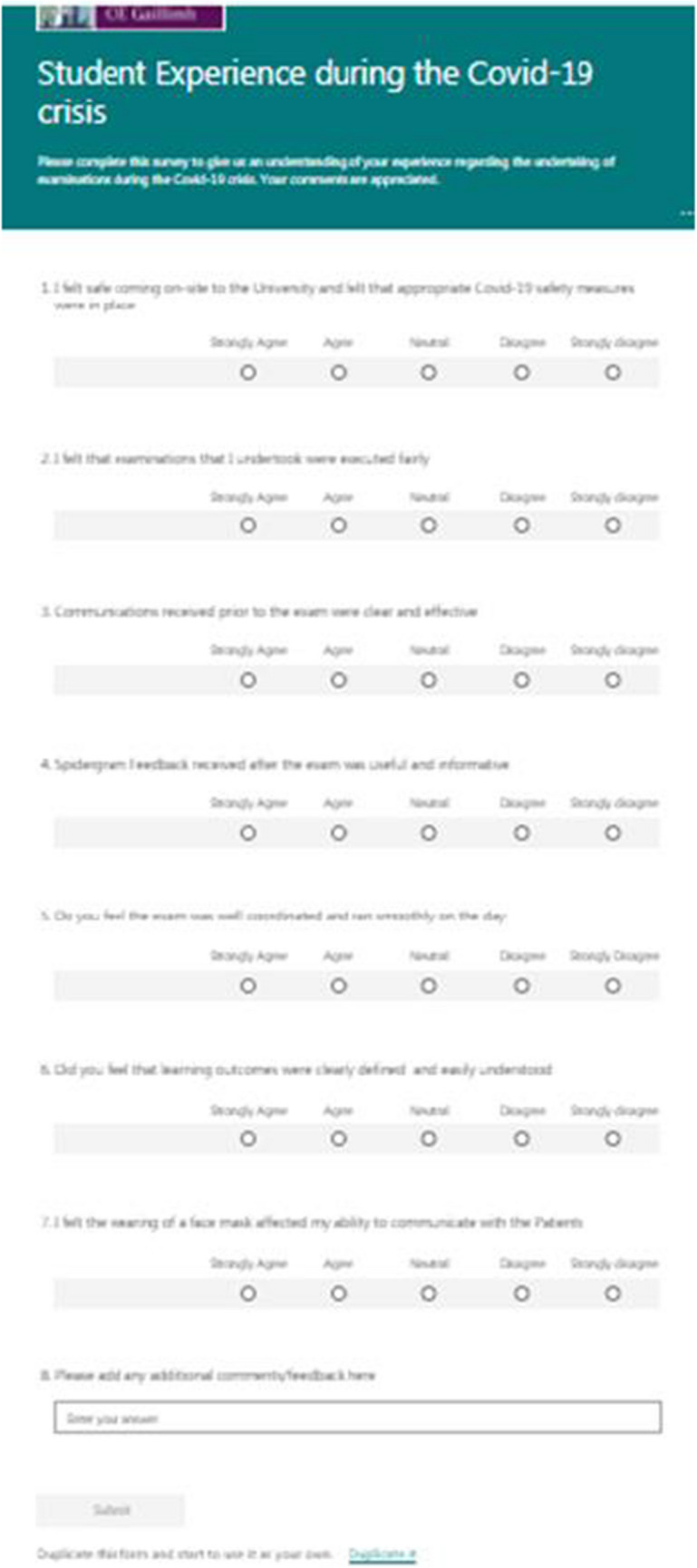
Student survey 2020 and 2021

## Data Availability

The data that support the findings of this study are available in anonymised form from the corresponding author, [AOM], upon reasonable request.

## Appendix 1

See Table 5.

**Table 5:** Station format and changes instituted secondary to the COVID-19 pandemic

	2019	2020	2021
Stations			
• Number and Time	16 x 8 minutes	4 x 14 minutes	8 x 12 minutes
• Time-gap between Stations	1 minute	3 minutes	2 minutes
• Students per circuit	14	5	9
• Concurrent Circuits	3 (2 days)	6 (1 day)	6 (2 days)
Administrative Actions			
• Student Segregation	Students sequestered for 1 hour (same stations in morning and afternoon).	No students sequestered (i.e. each circuit had different station material).	Students sequestered for 1 hour (same stations in morning and afternoon).
• Examiner Segregation at congregated breaks (i.e. lunch)	No	Yes (i.e. lunch packs provided in examiner rooms)	No
• Examiner Training	Discipline support pre-OSCE	Discipline support pre-OSCEOSCE stations examined by experienced examiners who had training by a “lead examiner” for each station prior to the OSCE.	Discipline support pre-OSCEOSCE stations examined by experienced examiners who had training by a “lead examiner” for each station prior to the OSCE.
Public health measures			
• Face masks	N/A	Examiners, students, administrative staff and simulated patients	Examiners, students, administrative staff and simulated patients
• Temperature checks at exam site prior to OSCE	N/A	Examiners, students, administrative staff and simulated patients	No temperature checks
• Health declaration regarding symptoms of COVID-19	N/A	Examiners, students, administrative staff and simulated patients – signed declaration of no symptoms of COVID-19	Examiners, students, administrative staff and simulated patients – advised not to attend if symptoms of COVID-19
• Hand Sanitation guidelines	N/A	Strict regulations	Strict regulations
• Travel declarations	N/A	Examiners, students, administrators and simulated patients if recently travelled outside Ireland.	No
• Social distancing and OSCE stations		Alteration of some clinical stations to ensure social distancing, with no clinical stations requiring physical examination of a person – mannequins utilised instead.	No alterations to clinical stations

## Appendix 2

See Fig. 2..
